# Crystal structures of 1-(4-chloro­phen­yl)-2-(di­phenyl­phosphor­yl)ethan-1-one and 1-(di­phenyl­phosphor­yl)-3,3-di­methyl­butan-2-one

**DOI:** 10.1107/S2056989015006994

**Published:** 2015-04-22

**Authors:** Erin G. Leach, Alyssa A. Kulesza, Richard J. Staples, Shannon M. Biros

**Affiliations:** a Department of Chemistry, Grand Valley State University, 1 Campus Dr., Allendale, MI 49401, USA; b Center for Crystallographic Research, Department of Chemistry, Michigan State University, 578 S. Shaw Lane, East Lansing, MI 48824, USA

**Keywords:** crystal structure, carbamoyl­methyl­phosphane oxide (CMPO), α-bromo­ketone, isopropoxydi­phenyl­phosphane, C—H⋯O hydrogen bonds, C—H⋯π inter­actions

## Abstract

The title compounds, C_20_H_16_ClO_2_P, (I), and C_18_H_21_O_2_P, (II), were synthesized *via* an Arbuzov reaction between an α-bromo­ketone and isopropoxydi­phenyl­phosphane. In the crystals of both compounds, mol­ecules are linked *via* bifurcated C—H⋯(O,O) hydrogen bonds, forming chains propagating along [100] for (I) and along [010] for (II). The chains are linked *via* C—H⋯π inter­actions, leading to the formation of sheets lying parallel to (010) for (I) and (001) for (II). The absolute structure of compound (II) was determined by resonant scattering [Flack parameter = 0.088 (14)].

## Abstract 2

The title compounds were synthesized *via* an Arbuzov reaction between an α-bromo­ketone and isopropoxydi­phenyl­phosphane. In the crystals of both compounds, mol­ecules are linked *via* bifurcated C—H⋯(O,O) hydrogen bonds, forming chains propagating along [100] and along [010].

## Chemical context   

The luminescent properties of lanthanide metals continue to gain attention from researchers inter­ested in the coordination chemistry of *f*-block elements. Direct excitation of lanthanides is difficult due to the parity forbidden *f*–*f* transitions required and relatively low molar absorptivities, but fortunately this excitation can be sensitized with an appropriate organic ligand. The ligand acts as an antenna by harvesting the excitation energy and transferring this energy to the metal emitting state (Weissman, 1942 ▸). The resulting emission bands have peak widths less than 10 nm, with a color characteristic of each lanthanide ion. As such, lanthanide metals have found uses in both material and biological applications (de Bettencourt-Dias, 2007 ▸; Thibon & Pierre, 2009 ▸; Eliseeva & Bünzli, 2010 ▸).
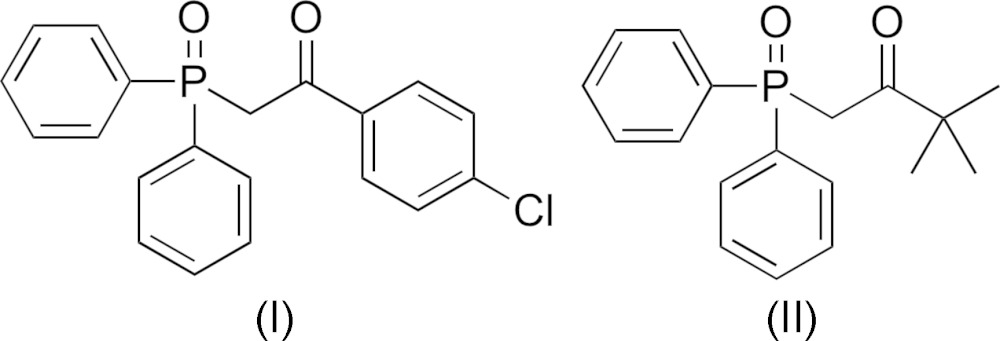


Recently, the carbamoyl­methyl­phosphane oxide (CMPO) group has been shown to be an effective ligand for the sensitization of lanthanide luminescence (Sharova *et al.*, 2012 ▸; Rosario-Amorin *et al.*, 2013 ▸; Sartain *et al.*, 2015 ▸). We undertook this work to investigate the role of the aryl carbonyl group on the ability of the CMPO moiety to act as an antenna in this process. Tuning the structure of these organic ligands may be tantamount to potential improvements in the absorption, transfer, and emission of energy by the resultant lanthanide–ligand complex. We report herein on the synthesis and crystal structure of two new CMPO ligands.

## Structural commentary   

**Figure 1:**
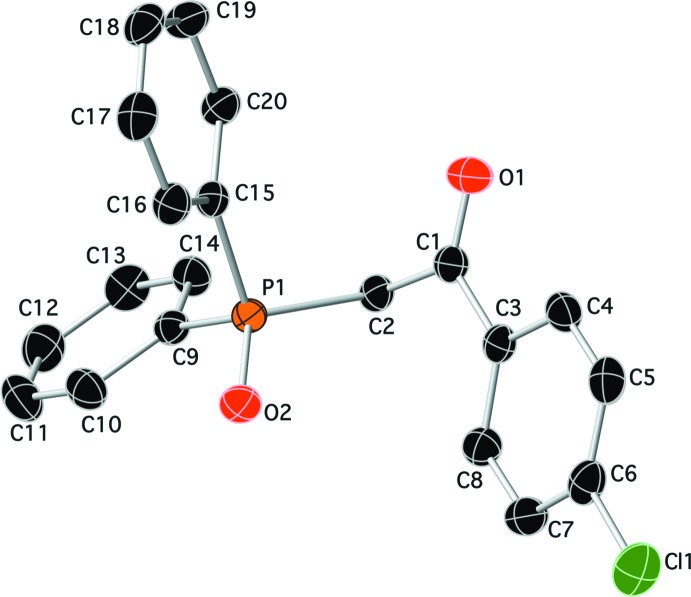
A view of the mol­ecular structure of compound (I), showing the atom labelling. Displacement ellipsoids are drawn at the 50% probability level. H atoms have been omitted for clarity.

The mol­ecular structures of compounds (I) and (II) are shown in Figs. 1 ▸ and 2 ▸, respectively. While compound (I) crystallized in the ortho­rhom­bic centrosymmetric space group *Pbca*, compound (II) crystallized in the chiral monoclinic space group *P*2_1_. In compound (I), the two phenyl rings (C9–C14 and C15–C20) are inclined to one another by 75.53 (8)°, and to the chloro­benzene ring (C3–C8) by 47.98 (8) and 62.16 (8)°, respectively. Atom P1 has a distorted tetra­hedral geometry with the C—P=O bond angles varying from 112.02 (7) to 114.35 (7)°, while the C—P—C angles vary from 105.04 (7) to 106.60 (7)°. The carbonyl group (C1=O1) and the phosphoryl group (P1=O2) are *anti* to one another, most probably to minimize unfavourable dipole–dipole inter­actions. In compound (II), the two phenyl rings (C7–C12 and C13–C18) are inclined to one another by 86.4 (2)°. Atom P1 also has a distorted tetra­hedral geometry with the C—P=O bond angles varying from 111.47 (16) to 115.06 (16)°, while the C—P—C bond angles vary from 101.84 (15) to 109.21 (16)°. Here the carbonyl group (C1=O1) and the phosphoryl group (P1=O2) are *syn* to one another.

## Supra­molecular features   

**Figure 3:**
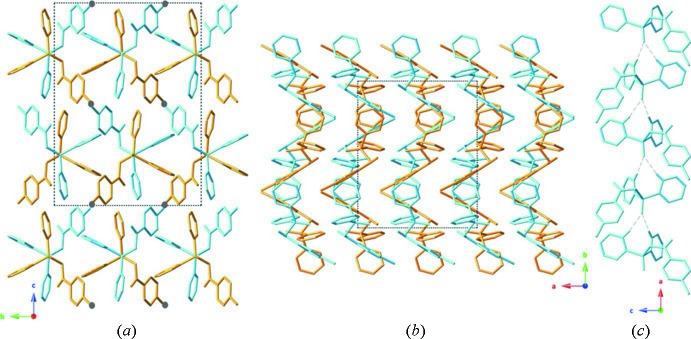
The crystal packing diagram of compound (I) (drawn as blue and orange sticks) viewed along: (*a*) the *a* axis (the Cl atoms are shown as dark grey dots); (*b*) the *c* axis; (*c*) along the *b* axis, with the bifurcated hydrogen bonds shown as dashed lines (see Table 1 ▸ for details). H atoms have been omitted for clarity in parts (*a*) and (*b*) and only those involved in hydrogen bonding are shown in part (*c*).

In the crystal of (I), the phosphoryl groups are aligned with the *a* axis, and as the individual mol­ecules stack in this direction they appear to rotate around the chlorine atom that lies close to the twofold screw axis, creating a pinwheel arrangement of mol­ecules (Fig. 3 ▸). The mol­ecules are linked *via* bifurcated C—H⋯(O,O) hydrogen bonds, forming chains propagating along [100]; see Fig. 3 ▸ and Table 1 ▸. The chains are linked *via* C—H⋯π inter­actions (Table 1 ▸), forming sheets lying parallel to (010).

**Figure 4:**
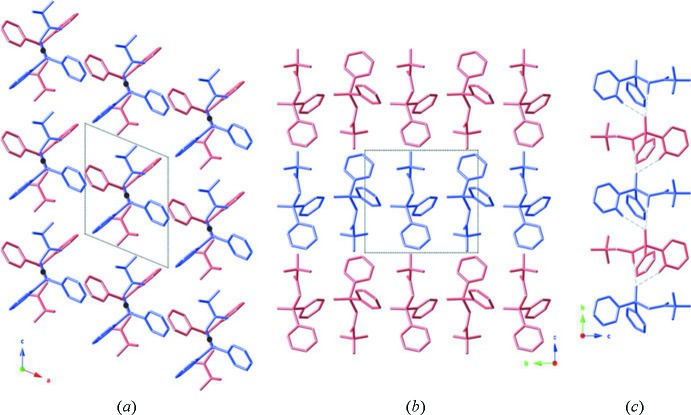
The crystal packing diagram of compound (II) (drawn as purple and pink sticks) viewed along: (*a*) the *b* axis (the center of the P1—C1 bond that coincides with the twofold screw axis is denoted with a grey dot); (*b*) the *a* axis; (*c*) along the *b* axis with the bifurcated hydrogen bonds shown as dashed lines (see Table 2 ▸ for details). H atoms have been omitted for clarity in parts (*a*) and (*b*) and only those involved in hydrogen bonding are shown in (*c*).

Compound (II) packs in a similar arrangement to (I) in the solid state, although subtle differences result in the formation of a chiral crystal from an achiral compound (Fig. 4 ▸). For compound (II), the phosphoryl groups are again aligned in one direction (along the *b* axis), but in this case, the P1—C2 bond in the center of the mol­ecule lies about a twofold screw axis and acts as the pivot point for the pinwheel arrangement rather than the terminal chlorine atom as seen in the crystal of compound (I). The absence of an inversion center or mirror plane results in a chiral twist to the packing within this crystal. Here, mol­ecules are also linked *via* bifurcated C—H⋯(O,O) hydrogen bonds, forming chains propagating along [010] (see Table 2 ▸ and Fig. 4 ▸) and the chains are linked *via* C—H⋯π inter­actions (Table 2 ▸), forming sheets parallel to (001).

## Database Survey   

The Cambridge Structural Database (CSD, Version 5.36, November 2014; Groom & Allen, 2014 ▸) contains 11 structures with a β-ketodi­phenyl­phosphoryl moiety. Three of these structures are related to the title compounds, but have either an alkyl group bonded to the keto function or branching at the α-carbon, *viz. E*-(5*SR*,6*SR*)-3,6-dimethyl-5-di­phenyl­phos­phinoyl-7-tri­phenyl­meth­oxy­hept-2-en-4-one acetone solvate (SUGWOG; Doyle *et al.*, 1993 ▸), anti-(2*S*,4*S*)-2-(*N*,*N*-dibenzyl­amino)-4-di­phenyl­phosphinoyl-1-phenyl­pentan-3-one monohydrate (RIZCEI; O’Brien *et al.*, 1997 ▸) and (4*R*,5*R*)-4,5-dihy­droxy-1,5-diphenyl-2-(di­phenyl­phosphino­yl)pentan-1-one) (FODBUW: Boesen *et al.*, 2005 ▸). The last compound (FODBUW) crystallizes in a chiral space group (*P*2_1_2_1_2_1_), as does compound (II). The phenyl rings of the di­phenyl­phosphinoyl group in each of these three compounds are inclined to one another by *ca* 67.97, 73.25 and 68.24°, respectively, similar to the arrangement in compound (I).

## Synthesis and crystallization   

The title compounds, (I) and (II), were prepared following slightly modified literature procedures (Arnaud-Neu *et al.*, 1996 ▸; Schuster *et al.*, 2009 ▸) by the Arbuzov reaction of isopropoxydi­phenyl­phosphane (Shintou *et al.*, 2003 ▸) with 2-bromo-4′-chloro­aceto­phenone for (I) and 1-bromo­pinacolone for (II). For both compounds, crystals suitable for X-ray diffraction analysis were grown by slow evaporation of a solution of the compound in CDCl_3_.

## Refinement   

Crystal data, data collection and structure refinement details are summarized in Table 3 ▸. The hydrogen atoms were placed in calculated positions and refined as riding atoms: C—H = 0.95–0.99 Å with *U*_iso_(H)= 1.5*U*_eq_(C) for methyl H atoms and 1.2*U*_eq_(C) for other H atoms.

## Supplementary Material

Crystal structure: contains datablock(s) global, I, II. DOI: 10.1107/S2056989015006994/su5111sup1.cif

Structure factors: contains datablock(s) I. DOI: 10.1107/S2056989015006994/su5111Isup2.hkl

Structure factors: contains datablock(s) II. DOI: 10.1107/S2056989015006994/su5111IIsup3.hkl

Supporting information file. DOI: 10.1107/S2056989015006994/su5111Isup4.cml

Supporting information file. DOI: 10.1107/S2056989015006994/su5111IIsup5.cml

CCDC references: 1058397, 1012828

Additional supporting information:  crystallographic information; 3D view; checkCIF report

## Figures and Tables

**Figure 2:**
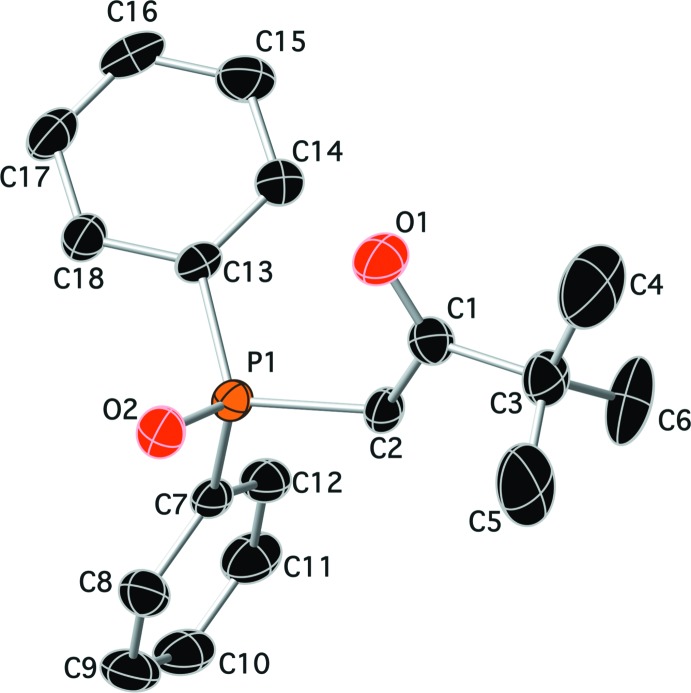
A view of the mol­ecular structure of compound (II), showing the atom labelling. Displacement ellipsoids are drawn at the 50% probability level. H atoms have been omitted for clarity.

**Table 1:** Hydrogen-bond geometry (Å, °) for (I) *Cg*1 and *Cg*3 are the centroids of rings C3–C8 and C15–C20, respectively.

*D*—H⋯*A*	*D*—H	H⋯*A*	*D*⋯*A*	*D*—H⋯*A*
C14—H14⋯O2^i^	0.95	2.30	3.1899 (19)	156
C20—H20⋯O2^i^	0.95	2.50	3.4487 (19)	176
C5—H5⋯*Cg*3^ii^	0.95	3.00	3.8873 (17)	156
C13—H13⋯*Cg*1^i^	0.95	2.90	3.5373 (19)	126

Symmetry codes: (i) 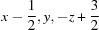; (ii) 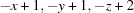.

**Table 2:** Hydrogen-bond geometry (Å, °) for (II) *Cg*1 is the centroid of ring C7–C12.

*D*—H⋯*A*	*D*—H	H⋯*A*	*D*⋯*A*	*D*—H⋯*A*
C2—H2*B*⋯O2^i^	0.99	2.19	3.176 (5)	176
C12—H12⋯O2^i^	0.95	2.53	3.373 (5)	148
C17—H17⋯*Cg*1^ii^	0.95	2.80	3.721 (5)	164

Symmetry codes: (i) 
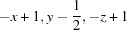
; (ii) 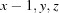.

**Table 3:** Experimental details

	(I)	(II)
Crystal data		
Chemical formula	C_20_H_16_ClO_2_P	C_18_H_21_O_2_P
*M*_r_	354.75	300.32
Crystal system, space group	Orthorhombic, *P* *b* *c* *a*	Monoclinic, *P*2_1_
Temperature (K)	173	173
*a*, *b*, *c* (Å)	11.7380 (2), 14.4453 (3), 19.9515 (3)	8.3416 (2), 10.5161 (2), 10.2790 (2)
α, β, γ (°)	90, 90, 90	90, 112.212 (1), 90
*V* (Å^3^)	3382.95 (10)	834.77 (3)
*Z*	8	2
Radiation type	Cu *K*α	Cu *K*α
μ (mm^−1^)	2.97	1.47
Crystal size (mm)	0.36 × 0.17 × 0.13	0.43 × 0.14 × 0.08

Data collection		
Diffractometer	Bruker APEXII CCD	Bruker SMART *APEX* CCD area detector
Absorption correction	Multi-scan (*SADABS*; Bruker, 2013 ▸)	Multi-scan (*SADABS*; Bruker, 2013 ▸)
*T*_min_, *T*_max_	0.599, 0.754	0.631, 0.754
No. of measured, independent and observed [*I* > 2σ(*I*)] reflections	17900, 3297, 2880	7043, 3006, 2774
*R*_int_	0.033	0.042
(sin θ/λ)_max_ (Å^−1^)	0.617	0.617

Refinement		
*R*[*F* ^2^ > 2σ(*F* ^2^)], *wR*(*F* ^2^), *S*	0.032, 0.089, 1.04	0.042, 0.118, 1.13
No. of reflections	3297	3006
No. of parameters	217	193
No. of restraints	0	1
H-atom treatment	H-atom parameters constrained	H-atom parameters constrained
Δρ_max_, Δρ_min_ (e Å^−3^)	0.32, −0.39	0.49, −0.38
Absolute structure	–	Flack *x* determined using 1090 quotients [(*I* ^+^)−(*I* ^−^)]/[(*I* ^+^)+(*I* ^−^)] (Parsons *et al.*, 2013 ▸)
Absolute structure parameter	–	0.088 (14)

Computer programs: *APEX2*, *SAINT* and *XPREP* (Bruker, 2013 ▸), *SHELXS97* (Sheldrick, 2008 ▸), *SHELXL2014* (Sheldrick, 2015 ▸), *CrystalMaker* (Palmer, 2007 ▸) and *OLEX2* (Dolomanov *et al.*, 2009 ▸; Bourhis *et al.*, 2015 ▸).

## supplementary crystallographic information

### (I) 1-(4-Chlorophenyl)-2-(diphenylphosphoryl)ethan-1-one Crystal data

**Table:**

C_20_H_16_ClO_2_P	*D*_x_ = 1.393 Mg m^−^^3^
*M**_r_* = 354.75	Cu *K*α radiation, λ = 1.54178 Å
Orthorhombic, *P**b**c**a*	Cell parameters from 9053 reflections
*a* = 11.7380 (2) Å	θ = 4.4–72.0°
*b* = 14.4453 (3) Å	µ = 2.97 mm^−^^1^
*c* = 19.9515 (3) Å	*T* = 173 K
*V* = 3382.95 (10) Å^3^	Needle, colourless
*Z* = 8	0.36 × 0.17 × 0.13 mm
*F*(000) = 1472	

### (I) 1-(4-Chlorophenyl)-2-(diphenylphosphoryl)ethan-1-one Data collection

**Table:**

Bruker APEXII CCD diffractometer	2880 reflections with *I* > 2σ(*I*)
Graphite monochromator	*R*_int_ = 0.033
φ and ω scans	θ_max_ = 72.2°, θ_min_ = 4.4°
Absorption correction: multi-scan (SADABS; Bruker, 2013)	*h* = −14→14
*T*_min_ = 0.599, *T*_max_ = 0.754	*k* = −17→17
17900 measured reflections	*l* = −24→23
3297 independent reflections	

### (I) 1-(4-Chlorophenyl)-2-(diphenylphosphoryl)ethan-1-one Refinement

**Table:**

Refinement on *F*^2^	Primary atom site location: structure-invariant direct methods
Least-squares matrix: full	Hydrogen site location: inferred from neighbouring sites
*R*[*F*^2^ > 2σ(*F*^2^)] = 0.032	H-atom parameters constrained
*wR*(*F*^2^) = 0.089	*w* = 1/[σ^2^(*F*_o_^2^) + (0.0502*P*)^2^ + 1.0258*P*] where *P* = (*F*_o_^2^ + 2*F*_c_^2^)/3
*S* = 1.04	(Δ/σ)_max_ = 0.001
3297 reflections	Δρ_max_ = 0.32 e Å^−^^3^
217 parameters	Δρ_min_ = −0.39 e Å^−^^3^
0 restraints	

### (I) 1-(4-Chlorophenyl)-2-(diphenylphosphoryl)ethan-1-one Special details

**Table:**

Geometry. All e.s.d.'s (except the e.s.d. in the dihedral angle between two l.s. planes) are estimated using the full covariance matrix. The cell e.s.d.'s are taken into account individually in the estimation of e.s.d.'s in distances, angles and torsion angles; correlations between e.s.d.'s in cell parameters are only used when they are defined by crystal symmetry. An approximate (isotropic) treatment of cell e.s.d.'s is used for estimating e.s.d.'s involving l.s. planes.

### (I) 1-(4-Chlorophenyl)-2-(diphenylphosphoryl)ethan-1-one Fractional atomic coordinates and isotropic or equivalent isotropic displacement parameters (Å^2^)

**Table:**

	*x*	*y*	*z*	*U*_iso_*/*U*_eq_	
Cl1	0.80844 (4)	0.25495 (3)	0.98830 (2)	0.04355 (14)	
P1	0.47545 (3)	0.53879 (3)	0.75031 (2)	0.02092 (11)	
O1	0.33462 (10)	0.47690 (9)	0.89963 (6)	0.0366 (3)	
O2	0.60062 (9)	0.52376 (8)	0.75506 (6)	0.0292 (3)	
C1	0.40409 (13)	0.43971 (11)	0.86325 (8)	0.0257 (3)	
C2	0.38970 (12)	0.44712 (10)	0.78795 (7)	0.0243 (3)	
H2A	0.3084	0.4587	0.7777	0.029*	
H2B	0.4109	0.3873	0.7673	0.029*	
C3	0.50328 (13)	0.38850 (10)	0.89190 (7)	0.0252 (3)	
C4	0.51562 (14)	0.38806 (11)	0.96164 (8)	0.0293 (3)	
H4	0.4594	0.4170	0.9887	0.035*	
C5	0.60803 (15)	0.34632 (12)	0.99183 (8)	0.0328 (4)	
H5	0.6163	0.3468	1.0392	0.039*	
C6	0.68856 (14)	0.30356 (11)	0.95145 (9)	0.0314 (4)	
C7	0.67686 (14)	0.30005 (12)	0.88247 (9)	0.0324 (4)	
H7	0.7316	0.2685	0.8559	0.039*	
C8	0.58415 (14)	0.34315 (11)	0.85264 (8)	0.0289 (3)	
H8	0.5758	0.3417	0.8053	0.035*	
C9	0.42652 (13)	0.54676 (10)	0.66510 (7)	0.0236 (3)	
C10	0.50496 (14)	0.57592 (12)	0.61691 (8)	0.0319 (4)	
H10	0.5815	0.5886	0.6293	0.038*	
C11	0.47052 (16)	0.58623 (13)	0.55074 (9)	0.0391 (4)	
H11	0.5234	0.6071	0.5180	0.047*	
C12	0.35988 (17)	0.56633 (13)	0.53235 (8)	0.0384 (4)	
H12	0.3372	0.5726	0.4869	0.046*	
C13	0.28230 (15)	0.53745 (12)	0.57968 (9)	0.0347 (4)	
H13	0.2063	0.5238	0.5667	0.042*	
C14	0.31441 (13)	0.52817 (11)	0.64629 (8)	0.0283 (3)	
H14	0.2603	0.5092	0.6789	0.034*	
C15	0.43163 (12)	0.64440 (10)	0.79158 (7)	0.0230 (3)	
C16	0.51476 (14)	0.70315 (11)	0.81674 (8)	0.0290 (3)	
H16	0.5927	0.6859	0.8143	0.035*	
C17	0.48423 (15)	0.78728 (12)	0.84549 (8)	0.0338 (4)	
H17	0.5413	0.8276	0.8624	0.041*	
C18	0.37069 (16)	0.81216 (12)	0.84948 (8)	0.0345 (4)	
H18	0.3498	0.8701	0.8683	0.041*	
C19	0.28765 (15)	0.75256 (12)	0.82598 (9)	0.0345 (4)	
H19	0.2097	0.7692	0.8299	0.041*	
C20	0.31663 (13)	0.66892 (11)	0.79674 (8)	0.0286 (3)	
H20	0.2591	0.6286	0.7803	0.034*	

### (I) 1-(4-Chlorophenyl)-2-(diphenylphosphoryl)ethan-1-one Atomic displacement parameters (Å^2^)

**Table:**

	*U*^11^	*U*^22^	*U*^33^	*U*^12^	*U*^13^	*U*^23^
Cl1	0.0357 (2)	0.0428 (3)	0.0521 (3)	0.00128 (18)	−0.01410 (19)	0.00816 (19)
P1	0.0163 (2)	0.0199 (2)	0.0265 (2)	−0.00157 (13)	−0.00033 (13)	−0.00008 (13)
O1	0.0337 (6)	0.0397 (7)	0.0364 (6)	0.0080 (5)	0.0075 (5)	−0.0014 (5)
O2	0.0181 (5)	0.0310 (6)	0.0386 (6)	−0.0002 (5)	−0.0015 (4)	0.0010 (5)
C1	0.0251 (7)	0.0207 (7)	0.0311 (8)	−0.0041 (6)	0.0033 (6)	−0.0002 (6)
C2	0.0215 (7)	0.0207 (7)	0.0307 (8)	−0.0029 (6)	−0.0001 (6)	0.0011 (6)
C3	0.0264 (7)	0.0203 (7)	0.0289 (7)	−0.0046 (6)	0.0017 (6)	0.0012 (6)
C4	0.0330 (8)	0.0259 (8)	0.0292 (8)	−0.0031 (7)	0.0059 (6)	0.0006 (6)
C5	0.0416 (9)	0.0299 (9)	0.0269 (8)	−0.0052 (7)	−0.0015 (7)	0.0036 (6)
C6	0.0291 (8)	0.0255 (8)	0.0395 (9)	−0.0037 (7)	−0.0057 (7)	0.0052 (7)
C7	0.0303 (8)	0.0305 (9)	0.0365 (9)	0.0029 (7)	0.0014 (7)	−0.0023 (7)
C8	0.0314 (8)	0.0279 (8)	0.0275 (7)	0.0008 (6)	−0.0002 (6)	−0.0012 (6)
C9	0.0231 (7)	0.0207 (7)	0.0269 (7)	−0.0003 (6)	0.0003 (6)	−0.0011 (5)
C10	0.0281 (8)	0.0333 (9)	0.0342 (8)	−0.0055 (7)	0.0053 (7)	−0.0028 (7)
C11	0.0454 (10)	0.0409 (10)	0.0310 (8)	−0.0048 (8)	0.0106 (7)	0.0015 (7)
C12	0.0508 (11)	0.0371 (10)	0.0273 (8)	0.0020 (8)	−0.0037 (7)	0.0012 (7)
C13	0.0328 (9)	0.0356 (10)	0.0357 (9)	−0.0013 (7)	−0.0088 (7)	−0.0015 (7)
C14	0.0241 (8)	0.0299 (8)	0.0308 (8)	−0.0027 (6)	−0.0008 (6)	0.0011 (6)
C15	0.0242 (7)	0.0207 (7)	0.0240 (7)	−0.0016 (6)	0.0001 (6)	0.0007 (5)
C16	0.0277 (8)	0.0277 (8)	0.0315 (8)	−0.0037 (6)	−0.0027 (6)	−0.0001 (6)
C17	0.0440 (9)	0.0269 (9)	0.0307 (8)	−0.0075 (7)	−0.0060 (7)	−0.0030 (6)
C18	0.0511 (10)	0.0234 (8)	0.0291 (8)	0.0056 (7)	−0.0006 (7)	−0.0021 (6)
C19	0.0341 (9)	0.0302 (9)	0.0393 (9)	0.0088 (7)	0.0007 (7)	−0.0002 (7)
C20	0.0258 (7)	0.0256 (8)	0.0345 (8)	0.0008 (6)	−0.0026 (6)	0.0008 (6)

### (I) 1-(4-Chlorophenyl)-2-(diphenylphosphoryl)ethan-1-one Geometric parameters (Å, º)

**Table:**

Cl1—C6	1.7360 (16)	C9—C14	1.395 (2)
P1—O2	1.4882 (11)	C10—H10	0.9500
P1—C2	1.8251 (15)	C10—C11	1.389 (2)
P1—C9	1.7982 (15)	C11—H11	0.9500
P1—C15	1.8083 (15)	C11—C12	1.380 (3)
O1—C1	1.2168 (19)	C12—H12	0.9500
C1—C2	1.515 (2)	C12—C13	1.377 (3)
C1—C3	1.493 (2)	C13—H13	0.9500
C2—H2A	0.9900	C13—C14	1.388 (2)
C2—H2B	0.9900	C14—H14	0.9500
C3—C4	1.399 (2)	C15—C16	1.387 (2)
C3—C8	1.394 (2)	C15—C20	1.399 (2)
C4—H4	0.9500	C16—H16	0.9500
C4—C5	1.380 (2)	C16—C17	1.391 (2)
C5—H5	0.9500	C17—H17	0.9500
C5—C6	1.387 (2)	C17—C18	1.383 (3)
C6—C7	1.384 (2)	C18—H18	0.9500
C7—H7	0.9500	C18—C19	1.382 (3)
C7—C8	1.388 (2)	C19—H19	0.9500
C8—H8	0.9500	C19—C20	1.384 (2)
C9—C10	1.396 (2)	C20—H20	0.9500
			
O2—P1—C2	114.35 (7)	C14—C9—C10	119.68 (14)
O2—P1—C9	112.64 (7)	C9—C10—H10	120.2
O2—P1—C15	112.02 (7)	C11—C10—C9	119.67 (16)
C9—P1—C2	105.04 (7)	C11—C10—H10	120.2
C9—P1—C15	106.60 (7)	C10—C11—H11	119.9
C15—P1—C2	105.54 (7)	C12—C11—C10	120.30 (16)
O1—C1—C2	119.05 (14)	C12—C11—H11	119.9
O1—C1—C3	120.86 (14)	C11—C12—H12	119.9
C3—C1—C2	120.09 (13)	C13—C12—C11	120.22 (16)
P1—C2—H2A	108.9	C13—C12—H12	119.9
P1—C2—H2B	108.9	C12—C13—H13	119.8
C1—C2—P1	113.43 (10)	C12—C13—C14	120.44 (16)
C1—C2—H2A	108.9	C14—C13—H13	119.8
C1—C2—H2B	108.9	C9—C14—H14	120.2
H2A—C2—H2B	107.7	C13—C14—C9	119.69 (15)
C4—C3—C1	117.63 (14)	C13—C14—H14	120.2
C8—C3—C1	123.27 (14)	C16—C15—P1	118.74 (12)
C8—C3—C4	119.09 (15)	C16—C15—C20	119.81 (15)
C3—C4—H4	119.4	C20—C15—P1	121.42 (12)
C5—C4—C3	121.18 (15)	C15—C16—H16	119.9
C5—C4—H4	119.4	C15—C16—C17	120.17 (15)
C4—C5—H5	120.8	C17—C16—H16	119.9
C4—C5—C6	118.47 (15)	C16—C17—H17	120.0
C6—C5—H5	120.8	C18—C17—C16	119.95 (15)
C5—C6—Cl1	119.11 (13)	C18—C17—H17	120.0
C7—C6—Cl1	119.12 (13)	C17—C18—H18	120.1
C7—C6—C5	121.75 (15)	C19—C18—C17	119.88 (16)
C6—C7—H7	120.4	C19—C18—H18	120.1
C6—C7—C8	119.20 (15)	C18—C19—H19	119.6
C8—C7—H7	120.4	C18—C19—C20	120.89 (16)
C3—C8—H8	119.9	C20—C19—H19	119.6
C7—C8—C3	120.25 (15)	C15—C20—H20	120.4
C7—C8—H8	119.9	C19—C20—C15	119.27 (15)
C10—C9—P1	117.37 (12)	C19—C20—H20	120.4
C14—C9—P1	122.92 (12)		
			
Cl1—C6—C7—C8	−176.61 (13)	C4—C5—C6—Cl1	177.30 (13)
P1—C9—C10—C11	−177.86 (13)	C4—C5—C6—C7	−1.5 (3)
P1—C9—C14—C13	178.80 (13)	C5—C6—C7—C8	2.2 (3)
P1—C15—C16—C17	176.49 (12)	C6—C7—C8—C3	−0.7 (3)
P1—C15—C20—C19	−176.96 (12)	C8—C3—C4—C5	2.2 (2)
O1—C1—C2—P1	97.28 (15)	C9—P1—C2—C1	−170.57 (11)
O1—C1—C3—C4	−2.5 (2)	C9—P1—C15—C16	−117.67 (13)
O1—C1—C3—C8	178.73 (16)	C9—P1—C15—C20	60.42 (14)
O2—P1—C2—C1	65.44 (12)	C9—C10—C11—C12	−1.1 (3)
O2—P1—C9—C10	−25.46 (15)	C10—C9—C14—C13	1.0 (2)
O2—P1—C9—C14	156.68 (13)	C10—C11—C12—C13	1.0 (3)
O2—P1—C15—C16	5.96 (14)	C11—C12—C13—C14	0.1 (3)
O2—P1—C15—C20	−175.96 (12)	C12—C13—C14—C9	−1.1 (3)
C1—C3—C4—C5	−176.70 (15)	C14—C9—C10—C11	0.1 (2)
C1—C3—C8—C7	177.34 (15)	C15—P1—C2—C1	−58.14 (12)
C2—P1—C9—C10	−150.53 (13)	C15—P1—C9—C10	97.79 (13)
C2—P1—C9—C14	31.61 (15)	C15—P1—C9—C14	−80.08 (14)
C2—P1—C15—C16	130.99 (12)	C15—C16—C17—C18	0.5 (2)
C2—P1—C15—C20	−50.92 (14)	C16—C15—C20—C19	1.1 (2)
C2—C1—C3—C4	176.67 (14)	C16—C17—C18—C19	1.2 (3)
C2—C1—C3—C8	−2.1 (2)	C17—C18—C19—C20	−1.7 (3)
C3—C1—C2—P1	−81.86 (15)	C18—C19—C20—C15	0.6 (3)
C3—C4—C5—C6	−0.7 (2)	C20—C15—C16—C17	−1.6 (2)
C4—C3—C8—C7	−1.4 (2)		

### (I) 1-(4-Chlorophenyl)-2-(diphenylphosphoryl)ethan-1-one Hydrogen-bond geometry (Å, º)

Cg1 and Cg3 are the centroids of rings C3–C8 and C15–C20, respectively.

**Table:**

*D*—H···*A*	*D*—H	H···*A*	*D*···*A*	*D*—H···*A*
C14—H14···O2^i^	0.95	2.30	3.1899 (19)	156
C20—H20···O2^i^	0.95	2.50	3.4487 (19)	176
C5—H5···*Cg*3^ii^	0.95	3.00	3.8873 (17)	156
C13—H13···*Cg*1^i^	0.95	2.90	3.5373 (19)	126

Symmetry codes: (i) *x*−1/2, *y*, −*z*+3/2; (ii) −*x*+1, −*y*+1, −*z*+2.

### (II) 1-(Diphenylphosphoryl)-3,3-dimethylbutan-2-one Crystal data

**Table:**

C_18_H_21_O_2_P	*F*(000) = 320
*M**_r_* = 300.32	*D*_x_ = 1.195 Mg m^−^^3^
Monoclinic, *P*2_1_	Cu *K*α radiation, λ = 1.54178 Å
*a* = 8.3416 (2) Å	Cell parameters from 5567 reflections
*b* = 10.5161 (2) Å	θ = 4.7–72.0°
*c* = 10.2790 (2) Å	µ = 1.47 mm^−^^1^
β = 112.212 (1)°	*T* = 173 K
*V* = 834.77 (3) Å^3^	Needle, colourless
*Z* = 2	0.43 × 0.14 × 0.08 mm

### (II) 1-(Diphenylphosphoryl)-3,3-dimethylbutan-2-one Data collection

**Table:**

Bruker SMART APEX CCD area-detector diffractometer	3006 independent reflections
Radiation source: sealed tube	2774 reflections with *I* > 2σ(*I*)
Graphite monochromator	*R*_int_ = 0.042
Detector resolution: 8 pixels mm^-1^	θ_max_ = 72.0°, θ_min_ = 4.7°
ω and φ scans	*h* = −9→10
Absorption correction: multi-scan (SADABS; Bruker, 2013)	*k* = −12→12
*T*_min_ = 0.631, *T*_max_ = 0.754	*l* = −12→12
7043 measured reflections	

### (II) 1-(Diphenylphosphoryl)-3,3-dimethylbutan-2-one Refinement

**Table:**

Refinement on *F*^2^	Hydrogen site location: inferred from neighbouring sites
Least-squares matrix: full	H-atom parameters constrained
*R*[*F*^2^ > 2σ(*F*^2^)] = 0.042	*w* = 1/[σ^2^(*F*_o_^2^) + (0.0722*P*)^2^] where *P* = (*F*_o_^2^ + 2*F*_c_^2^)/3
*wR*(*F*^2^) = 0.118	(Δ/σ)_max_ < 0.001
*S* = 1.13	Δρ_max_ = 0.49 e Å^−^^3^
3006 reflections	Δρ_min_ = −0.38 e Å^−^^3^
193 parameters	Absolute structure: Flack *x* determined using 1090 quotients [(I+)-(I-)]/[(I+)+(I-)] (Parsons *et al.*, 2013)
1 restraint	Absolute structure parameter: 0.088 (14)
Primary atom site location: structure-invariant direct methods	

### (II) 1-(Diphenylphosphoryl)-3,3-dimethylbutan-2-one Special details

**Table:**

Geometry. All e.s.d.'s (except the e.s.d. in the dihedral angle between two l.s. planes) are estimated using the full covariance matrix. The cell e.s.d.'s are taken into account individually in the estimation of e.s.d.'s in distances, angles and torsion angles; correlations between e.s.d.'s in cell parameters are only used when they are defined by crystal symmetry. An approximate (isotropic) treatment of cell e.s.d.'s is used for estimating e.s.d.'s involving l.s. planes.

### (II) 1-(Diphenylphosphoryl)-3,3-dimethylbutan-2-one Fractional atomic coordinates and isotropic or equivalent isotropic displacement parameters (Å^2^)

**Table:**

	*x*	*y*	*z*	*U*_iso_*/*U*_eq_	
P1	0.46337 (9)	0.13395 (7)	0.54974 (7)	0.0247 (2)	
O1	0.2783 (3)	0.1230 (4)	0.2325 (3)	0.0450 (7)	
O2	0.4313 (3)	0.2739 (3)	0.5441 (3)	0.0352 (6)	
C1	0.4309 (5)	0.1015 (3)	0.2672 (3)	0.0328 (8)	
C2	0.5430 (4)	0.0741 (4)	0.4198 (3)	0.0286 (7)	
H2A	0.6592	0.1108	0.4399	0.034*	
H2B	0.5572	−0.0192	0.4317	0.034*	
C3	0.5199 (5)	0.0970 (4)	0.1613 (4)	0.0403 (9)	
C4	0.3850 (8)	0.0965 (11)	0.0140 (5)	0.105 (4)	
H4A	0.3118	0.0210	0.0006	0.157*	
H4B	0.4422	0.0952	−0.0536	0.157*	
H4C	0.3133	0.1731	−0.0008	0.157*	
C5	0.6294 (11)	0.2154 (7)	0.1830 (7)	0.090 (3)	
H5A	0.5596	0.2900	0.1842	0.135*	
H5B	0.6712	0.2237	0.1062	0.135*	
H5C	0.7284	0.2094	0.2726	0.135*	
C6	0.6318 (9)	−0.0220 (7)	0.1848 (6)	0.0713 (17)	
H6A	0.7288	−0.0159	0.2757	0.107*	
H6B	0.6765	−0.0295	0.1096	0.107*	
H6C	0.5619	−0.0970	0.1842	0.107*	
C7	0.6358 (4)	0.0881 (4)	0.7129 (3)	0.0280 (7)	
C8	0.7278 (5)	0.1814 (4)	0.8067 (4)	0.0400 (9)	
H8	0.6982	0.2684	0.7865	0.048*	
C9	0.8634 (5)	0.1477 (6)	0.9304 (4)	0.0518 (12)	
H9	0.9264	0.2117	0.9946	0.062*	
C10	0.9069 (5)	0.0207 (5)	0.9603 (4)	0.0485 (11)	
H10	0.9994	−0.0023	1.0449	0.058*	
C11	0.8157 (5)	−0.0721 (5)	0.8669 (4)	0.0459 (10)	
H11	0.8455	−0.1590	0.8873	0.055*	
C12	0.6802 (4)	−0.0389 (4)	0.7431 (4)	0.0341 (8)	
H12	0.6178	−0.1031	0.6790	0.041*	
C13	0.2784 (4)	0.0408 (4)	0.5431 (3)	0.0270 (6)	
C14	0.2209 (5)	−0.0666 (4)	0.4600 (4)	0.0399 (9)	
H14	0.2786	−0.0945	0.4012	0.048*	
C15	0.0789 (6)	−0.1332 (5)	0.4632 (5)	0.0486 (11)	
H15	0.0387	−0.2059	0.4051	0.058*	
C16	−0.0046 (5)	−0.0952 (5)	0.5495 (5)	0.0462 (10)	
H16	−0.1014	−0.1416	0.5513	0.055*	
C17	0.0532 (5)	0.0105 (4)	0.6328 (4)	0.0422 (9)	
H17	−0.0035	0.0366	0.6930	0.051*	
C18	0.1936 (5)	0.0793 (4)	0.6298 (4)	0.0337 (7)	
H18	0.2320	0.1528	0.6870	0.040*	

### (II) 1-(Diphenylphosphoryl)-3,3-dimethylbutan-2-one Atomic displacement parameters (Å^2^)

**Table:**

	*U*^11^	*U*^22^	*U*^33^	*U*^12^	*U*^13^	*U*^23^
P1	0.0238 (4)	0.0270 (4)	0.0251 (3)	−0.0011 (3)	0.0113 (3)	0.0003 (3)
O1	0.0312 (12)	0.068 (2)	0.0324 (11)	0.0044 (14)	0.0085 (10)	0.0033 (14)
O2	0.0398 (13)	0.0270 (14)	0.0428 (14)	0.0012 (10)	0.0200 (11)	0.0020 (10)
C1	0.0345 (17)	0.037 (2)	0.0268 (15)	−0.0019 (13)	0.0119 (13)	−0.0008 (13)
C2	0.0276 (15)	0.0364 (18)	0.0257 (15)	0.0004 (13)	0.0143 (12)	−0.0003 (13)
C3	0.043 (2)	0.054 (3)	0.0278 (16)	0.0021 (17)	0.0182 (16)	0.0016 (15)
C4	0.068 (3)	0.218 (12)	0.027 (2)	0.022 (5)	0.018 (2)	−0.004 (4)
C5	0.138 (6)	0.084 (5)	0.094 (5)	−0.045 (5)	0.096 (5)	−0.025 (4)
C6	0.092 (4)	0.080 (4)	0.067 (3)	0.029 (3)	0.059 (3)	0.011 (3)
C7	0.0222 (13)	0.0427 (18)	0.0212 (14)	−0.0043 (13)	0.0106 (12)	−0.0009 (12)
C8	0.0411 (19)	0.047 (2)	0.0348 (19)	−0.0138 (17)	0.0173 (16)	−0.0079 (15)
C9	0.0411 (19)	0.079 (3)	0.0333 (17)	−0.027 (2)	0.0120 (15)	−0.016 (2)
C10	0.0291 (17)	0.083 (3)	0.0281 (17)	−0.006 (2)	0.0045 (14)	0.0044 (19)
C11	0.0315 (18)	0.063 (3)	0.038 (2)	0.0070 (18)	0.0082 (16)	0.0108 (19)
C12	0.0267 (16)	0.043 (2)	0.0292 (17)	−0.0013 (14)	0.0066 (14)	0.0005 (14)
C13	0.0200 (13)	0.0328 (16)	0.0276 (15)	0.0006 (12)	0.0083 (12)	0.0047 (13)
C14	0.0336 (18)	0.042 (2)	0.047 (2)	−0.0061 (16)	0.0181 (17)	−0.0107 (16)
C15	0.0392 (19)	0.041 (3)	0.065 (3)	−0.012 (2)	0.019 (2)	−0.0121 (19)
C16	0.0262 (16)	0.052 (2)	0.062 (3)	−0.0053 (18)	0.0184 (18)	0.011 (2)
C17	0.0314 (17)	0.057 (3)	0.044 (2)	0.0000 (17)	0.0216 (16)	0.0036 (18)
C18	0.0292 (16)	0.0431 (19)	0.0306 (16)	0.0007 (15)	0.0133 (14)	−0.0011 (14)

### (II) 1-(Diphenylphosphoryl)-3,3-dimethylbutan-2-one Geometric parameters (Å, º)

**Table:**

P1—O2	1.493 (3)	C7—C12	1.389 (6)
P1—C2	1.814 (3)	C8—H8	0.9500
P1—C7	1.814 (3)	C8—C9	1.391 (6)
P1—C13	1.807 (3)	C9—H9	0.9500
O1—C1	1.207 (5)	C9—C10	1.387 (8)
C1—C2	1.520 (5)	C10—H10	0.9500
C1—C3	1.533 (5)	C10—C11	1.378 (7)
C2—H2A	0.9900	C11—H11	0.9500
C2—H2B	0.9900	C11—C12	1.390 (5)
C3—C4	1.507 (6)	C12—H12	0.9500
C3—C5	1.509 (7)	C13—C14	1.388 (6)
C3—C6	1.525 (7)	C13—C18	1.391 (5)
C4—H4A	0.9800	C14—H14	0.9500
C4—H4B	0.9800	C14—C15	1.387 (6)
C4—H4C	0.9800	C15—H15	0.9500
C5—H5A	0.9800	C15—C16	1.378 (7)
C5—H5B	0.9800	C16—H16	0.9500
C5—H5C	0.9800	C16—C17	1.375 (7)
C6—H6A	0.9800	C17—H17	0.9500
C6—H6B	0.9800	C17—C18	1.387 (5)
C6—H6C	0.9800	C18—H18	0.9500
C7—C8	1.387 (5)		
			
O2—P1—C2	115.06 (16)	H6B—C6—H6C	109.5
O2—P1—C7	111.47 (16)	C8—C7—P1	119.5 (3)
O2—P1—C13	113.25 (15)	C8—C7—C12	119.6 (3)
C7—P1—C2	101.84 (15)	C12—C7—P1	120.9 (3)
C13—P1—C2	109.21 (16)	C7—C8—H8	120.0
C13—P1—C7	104.98 (16)	C7—C8—C9	120.0 (4)
O1—C1—C2	120.6 (3)	C9—C8—H8	120.0
O1—C1—C3	122.4 (3)	C8—C9—H9	119.9
C2—C1—C3	117.0 (3)	C10—C9—C8	120.1 (4)
P1—C2—H2A	108.3	C10—C9—H9	119.9
P1—C2—H2B	108.3	C9—C10—H10	120.1
C1—C2—P1	116.1 (2)	C11—C10—C9	119.9 (4)
C1—C2—H2A	108.3	C11—C10—H10	120.1
C1—C2—H2B	108.3	C10—C11—H11	119.9
H2A—C2—H2B	107.4	C10—C11—C12	120.2 (4)
C4—C3—C1	109.6 (4)	C12—C11—H11	119.9
C4—C3—C5	109.4 (6)	C7—C12—C11	120.1 (4)
C4—C3—C6	109.7 (5)	C7—C12—H12	119.9
C5—C3—C1	107.3 (3)	C11—C12—H12	119.9
C5—C3—C6	110.7 (5)	C14—C13—P1	123.8 (3)
C6—C3—C1	110.1 (4)	C14—C13—C18	119.4 (3)
C3—C4—H4A	109.5	C18—C13—P1	116.8 (3)
C3—C4—H4B	109.5	C13—C14—H14	120.1
C3—C4—H4C	109.5	C15—C14—C13	119.7 (4)
H4A—C4—H4B	109.5	C15—C14—H14	120.1
H4A—C4—H4C	109.5	C14—C15—H15	119.6
H4B—C4—H4C	109.5	C16—C15—C14	120.8 (4)
C3—C5—H5A	109.5	C16—C15—H15	119.6
C3—C5—H5B	109.5	C15—C16—H16	120.3
C3—C5—H5C	109.5	C17—C16—C15	119.5 (4)
H5A—C5—H5B	109.5	C17—C16—H16	120.3
H5A—C5—H5C	109.5	C16—C17—H17	119.7
H5B—C5—H5C	109.5	C16—C17—C18	120.6 (4)
C3—C6—H6A	109.5	C18—C17—H17	119.7
C3—C6—H6B	109.5	C13—C18—H18	120.0
C3—C6—H6C	109.5	C17—C18—C13	120.0 (4)
H6A—C6—H6B	109.5	C17—C18—H18	120.0
H6A—C6—H6C	109.5		
			
P1—C7—C8—C9	−178.3 (3)	C3—C1—C2—P1	158.4 (3)
P1—C7—C12—C11	178.3 (3)	C7—P1—C2—C1	−177.5 (3)
P1—C13—C14—C15	179.1 (3)	C7—P1—C13—C14	−102.8 (3)
P1—C13—C18—C17	−178.4 (3)	C7—P1—C13—C18	75.6 (3)
O1—C1—C2—P1	−23.1 (5)	C7—C8—C9—C10	0.0 (6)
O1—C1—C3—C4	−11.3 (7)	C8—C7—C12—C11	0.3 (5)
O1—C1—C3—C5	107.4 (6)	C8—C9—C10—C11	0.1 (6)
O1—C1—C3—C6	−132.0 (5)	C9—C10—C11—C12	−0.1 (6)
O2—P1—C2—C1	−56.8 (3)	C10—C11—C12—C7	−0.1 (6)
O2—P1—C7—C8	−5.5 (3)	C12—C7—C8—C9	−0.2 (5)
O2—P1—C7—C12	176.4 (3)	C13—P1—C2—C1	71.8 (3)
O2—P1—C13—C14	135.4 (3)	C13—P1—C7—C8	−128.5 (3)
O2—P1—C13—C18	−46.2 (3)	C13—P1—C7—C12	53.5 (3)
C2—P1—C7—C8	117.7 (3)	C13—C14—C15—C16	−1.0 (7)
C2—P1—C7—C12	−60.4 (3)	C14—C13—C18—C17	0.0 (6)
C2—P1—C13—C14	5.8 (4)	C14—C15—C16—C17	0.3 (7)
C2—P1—C13—C18	−175.8 (3)	C15—C16—C17—C18	0.5 (7)
C2—C1—C3—C4	167.2 (5)	C16—C17—C18—C13	−0.7 (6)
C2—C1—C3—C5	−74.1 (5)	C18—C13—C14—C15	0.8 (6)
C2—C1—C3—C6	46.5 (5)		

### (II) 1-(Diphenylphosphoryl)-3,3-dimethylbutan-2-one Hydrogen-bond geometry (Å, º)

Cg1 is the centroid of ring C7–C12.

**Table:**

*D*—H···*A*	*D*—H	H···*A*	*D*···*A*	*D*—H···*A*
C2—H2*B*···O2^i^	0.99	2.19	3.176 (5)	176
C12—H12···O2^i^	0.95	2.53	3.373 (5)	148
C17—H17···*Cg*1^ii^	0.95	2.80	3.721 (5)	164

Symmetry codes: (i) −*x*+1, *y*−1/2, −*z*+1; (ii) *x*−1, *y*, *z*.
